# Calcium Binding to Beta-2-Microglobulin at Physiological Ph Drives the Occurrence of Conformational Changes Which Cause the Protein to Precipitate into Amorphous Forms That Subsequently Transform into Amyloid Aggregates

**DOI:** 10.1371/journal.pone.0095725

**Published:** 2014-04-22

**Authors:** Sukhdeep Kumar, Prerna Sharma, Kanika Arora, Manoj Raje, Purnananda Guptasarma

**Affiliations:** 1 Department of Biological Sciences, Indian Institute of Science Education and Research (IISER) Mohali, SAS Nagar, Punjab, India; 2 Council of Scientific and Industrial Research, Institute of Microbial Technology (CSIR-IMTECH), Chandigarh, India; INRA, France

## Abstract

Using spectroscopic, calorimetric and microscopic methods, we demonstrate that calcium binds to beta-2-microglobulin (β2m) under physiological conditions of pH and ionic strength, in biological buffers, causing a conformational change associated with the binding of up to four calcium atoms per β2m molecule, with a marked transformation of some random coil structure into beta sheet structure, and culminating in the aggregation of the protein at physiological (serum) concentrations of calcium and β2m. We draw attention to the fact that the sequence of β2m contains several potential calcium-binding motifs of the DXD and DXDXD (or DXEXD) varieties. We establish (a) that the microscopic aggregation seen at physiological concentrations of β2m and calcium turns into actual turbidity and visible precipitation at higher concentrations of protein and β2m, (b) that this initial aggregation/precipitation leads to the formation of amorphous aggregates, (c) that the formation of the amorphous aggregates can be partially reversed through the addition of the divalent ion chelating agent, EDTA, and (d) that upon incubation for a few weeks, the amorphous aggregates appear to support the formation of amyloid aggregates that bind to the dye, thioflavin T (ThT), resulting in increase in the dye's fluorescence. We speculate that β2m exists in the form of microscopic aggregates *in vivo* and that these don't progress to form larger amyloid aggregates because protein concentrations remain low under normal conditions of kidney function and β2m degradation. However, when kidney function is compromised and especially when dialysis is performed, β2m concentrations probably transiently rise to yield large aggregates that deposit in bone joints and transform into amyloids during dialysis related amyloidosis.

## Introduction

Human β2-microglobulin (β2m), also known as the MHC-I light chain, is a small protein constituent of all Class-I major histocompatibility (MHC-I) complexes displayed on the surfaces of human cells [1]. As a polypeptide chain which is only 99 residues in length, β2m manages to chaperone the folding of the much larger MHC-I heavy chain polypeptide within MHC-I complexes, which is known as the human leukocyte antigen, or HLA, chain. The binding and display of peptides to T-cell receptors by the HLA chain is critically dependent on the correctness of its assembly with β2m [2]. When the complex is disassembled during natural turnover, the non-covalently associated β2m molecule is thought to be simply ‘shed’ into extracellular fluids by the displaying cell, while the membrane-tethered HLA chain is internalized. The ‘shed’ β2m molecule is then carried to the kidney where it is degraded. This results in an equilibrium β2m concentration of ∼1–3 µg/ml in the serum of healthy humans. In patients suffering from renal dysfunction, the degradation of β2m in the kidney becomes compromised [3], leading to elevated β2m concentrations in the serum. Under such conditions, β2m levels can be as high as 25–60 times the concentrations seen in healthy humans [4]. An apparent consequence of these elevated concentrations is that β2m tends to aggregate and deposit as insoluble amyloid precipitates within the joints of patients receiving hemodialysis-based treatment. This leads to Dialysis Related Amyloidosis (DRA), a condition which includes carpal tunnel syndrome, amyloid arthopathy, and pathological bone disruption [5],[6]. There is much interest, therefore, in the aggregation and deposition of this small seven β-stranded (anti-parallel β-sandwich) protein.

One significant element of dissatisfaction with our current appreciation of DRA, however, is that the cause-effect relationship between elevated β2m concentrations, on the one hand, and β2m deposition, on the other, is not at all clear. As a protein, β2m is exceptionally soluble in aqueous solutions at physiological pH and ionic strength. Under these conditions, the protein displays no tendency to undergo aggregation; in fact, β2m can even be concentrated up to levels as high as several tens of milligrams per milliliter (i.e., millimolar concentrations) with no consequent aggregation. In fact, the protein can even be incubated for several months at such high concentrations, at 37 °C, in buffers of neutral pH, with no observable precipitation [6]–[9]. Notably, such concentrations are orders of magnitude higher than both (a) the elevated levels seen in DRA patients, and (b) the levels seen in healthy individuals.

Thus, elevated levels of β2m alone cannot explain DRA. Attempts have been made to create a mouse model for DRA, using transgenic mice that over-express human β2m to such high levels that serum concentrations exceed those seen in DRA patients by a factor of four. Yet such mice are neither found to be prone to develop DRA on their own, nor prone to develop DRA through the introduction of pre-existing β2m amyloid fibrils in the form of seeds [10]. It is not even as if differences in conformation between soluble β2m and HLA-bound β2m reveal very significant clues to its precipitation, because the molecule's solution structure and HLA-bound structure are very similar, with only minor changes in beta-strand composition and arrangement distinguishing the two structures, as reviewed [6]. Perhaps most intriguing of all is the fact that β2m displays the highest structural stability in solutions of physiological pH, of all pH values tested [11]. The precipitation and deposition of β2m under physiological conditions, at pH 7.4, thus continues to perplex those studying the molecule's behavior, and many papers discussing how such deposition occurs *in vivo* have failed to arrive at any definitive conclusions.

In the absence of clear insights into physiological deposition, the bulk of studies on β2m have focused instead on non-physiological conditions eliciting aggregation and precipitation. Thus, it is now well-known that β2m amyloids form quite readily under acidic conditions, requiring only a few weeks of incubation. Fibrils with different morphologies, lengths and twists tend to be observed under different acidic conditions, e.g., (i) long and straight fibrils are obtained in the pH range of 1.5–4.0 in buffers of ionic strength ≤50 mM, (ii) worm-like fibrils are obtained in the pH range of 2.5–4.0 in buffers of ionic strength ≥100 mM,while (iii) rod-like fibrils are obtained in the pH range of 3.0–4.0 in buffers of ionic strength ≥50 mM [6]. In addition to acidic pH, certain physical factors such as sonication [12], as well as chemicals such as glycosaminoglycan and proteoglycans [13]–[15], sodium dodecyl sulfate [16], collagen [17], [18], lysophosphatidic acid [19], [20], non-esterified fatty acids [21], and heparin [22], have been reported to lower the stability of β2m's native state at neutral pH, and also aid in the extension of amyloid fibrils. Therefore, much is now known about how β2m aggregates under non-physiological conditions. Much is also known about how the morphologies of its aggregated amyloid forms vary widely. A definitive understanding of physiological deposition, however, remains elusive.

Notably, some studies have focused on β2m oligomer formation at physiological pH and ionic strength in the presence of metal ions [23]–[27]. Miranker and colleagues [23], [24] have reported that Cu^2+^ binds specifically to β2m with a maximum stoichiometry of 4∶1 (as observed using LC-coupled ESI mass spectrometry), whereas other divalent ions bind either poorly and non-specifically, or not at all. Vachet and colleagues [25], [26] have reported that monomeric β2m binds Cu^2+^ via the N-terminal amine, the amide of Gln2, the imidazole ring of His31, and the carboxylate of Asp59, through a large conformational reorganization (relative to the HLA chain-bound conformation) which is important for establishing certain dimer-stabilizing salt bridges between Asp59 and Lys19. More importantly, the group has shown that when β2m is unfolded, up to four copper ions can bind.

Thus, copper binding by β2m has been observed and commented upon, and also held to be important for oligomer formation and DRA, although copper is only present in vanishingly low concentrations in the human body. It has been suggested that concentrations of copper might increase during dialysis due to the presence of copper in the equipment used for dialysis, leading to oligomerization, aggregation and precipitation of β2m [23]. However, this has not yet been established unequivocally [28].

Given the above, we engage here in revisiting the entire subject of metal binding by β2m. However, we are far more interested in the binding of β2m to calcium than to any other metal. There are several reasons for this. (i) Of all the divalent metal ions present in the human body, calcium is probably the one present in the serum in the highest concentration range (1–2 mM), with the largest number of known physiological, metabolic, biochemical and structural roles, causing it to be the metal with the highest potential degree of relevance to any disease involving metal binding; (ii) It is conceivable that those who have studied the binding of copper have not explored the binding of calcium sufficiently, owing to a preoccupation with copper. (iii) It is conceivable that different metal ions bind to β2m in somewhat different ways, such that the affinity or specificity of binding of one metal would not necessarily be entirely correlated with effects on protein conformation (if any), or on the propensity of the protein to aggregate and precipitate (if any) due to any other metal, with calcium being the most important metal to examine due to its significant physiological presence. (iv) There exists a little-noticed piece of work in the literature which indicates some sort of a cause-effect relationship between β2m and calcium, in respect of the behavior of calvariae. The protein, β2m, is thought to be mitogenic for both osteoblasts and osteoclasts. It has been reported that the addition of β2m to calvariae leads to a net efflux of calcium and osteoclast stimulation [29]. Although the report did not further investigate the mechanism by which this occurs, there are two possible explanations. On the one hand, it is possible that β2m binds to some cell surface proteins and induces the efflux of calcium. On the other hand, it is possible that there is a net ongoing influx-efflux of calcium which becomes affected by the presence of β2m as a titrant of calcium outside the cell, resulting in a net efflux of calcium. When this possibility is considered in the light of a separate report [30], which suggests that a negative correlation exists between β2m levels in the serum and the concentrations of free calcium in the serum, the link between calcium and β2m seems highly potentially significant.

We feel, therefore, that a strong case exists for examination of the direct binding (and sequestration) of calcium by β2m. Since there is not a single report in the literature to suggest either that such binding occurs quantitatively either *in vitro* or *in vivo*, or indeed that any such binding has effects upon β2m conformation or aggregation behavior [discounting the report in reference 23 that β2m binds to calcium non-specifically], we decided to examine whether β2m binds to calcium under physiological conditions.

**Figure 1:**
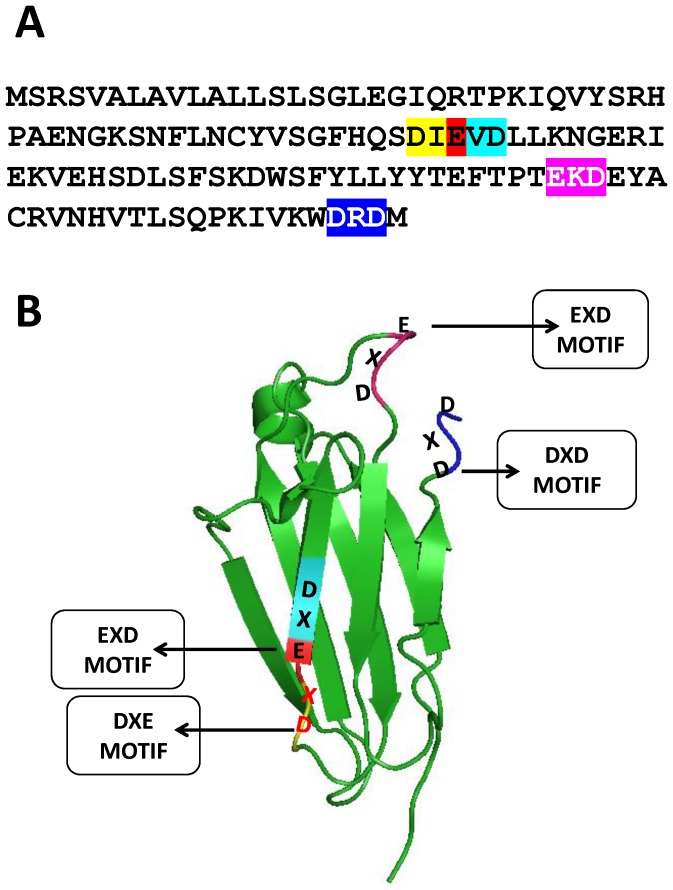
Potential additional/alternative sites for metal-binding and, in particular, calcium-binding in the sequence (Panel A) and structure (Panel B) of the HLA-interacting protein, β2m. Some metal-binding sites have already been noted in the literature, e.g., involving β2m's histidine residues, the N-terminal amino group, and some glutamine sidechains. The protein has also been reported to specifically bind to copper, but only non-specifically to calcium or zinc. In this paper, the focus is on calcium-binding. The DXDXD/DXDXE/DXEXD type of calcium-binding motif shown in the figure is found in microbial primases and topoisomerases; it is now quite well-known but its presence in β2m has not yet been noted in the literature. The DXD/DXE/EXD motif is even more well-known. It exists in glycosyl transferases and co-ordinately binds sugar and metal, but can also bind metal through both acidic sidechains.

We examined the structure and sequence of β2m in the light of the possible presence of metal binding sites other than those reported for copper. Figure 1A shows the sequence of β2m while Figure 1B shows a ribbon diagram representation of its beta sheet structure. From the figure, and in the light of what is known now about calcium-binding motifs, it is immediately evident that there are several potential motifs in β2m. It has been reported that DXD motifs in numerous enzymes participate in metal binding in association with sugar binding, through the involvement of the second aspartate residue in the DXD motif [31]–[33]. It has also been reported that, in certain instances, DXD motifs can even bind directly to metal atoms through both the aspartate side chains [34]. Further, in certain primase enzymes from thermophiles, and also in topoisomerases of various kinds, it has been reported that a DXDXD motif directly binds to calcium [35]. Allowing for substitutions of D by E, there appear to be either three or four sites containing sequences of one of the following varieties in the β2m sequence, as shown in Figure 1: DXD, DXE, EXD, DXDXD/DXDXE/DXEXD. We emphasize here that a protein with known roles in calcium efflux from cells and in the development of osteoclasts and osteoblasts in the bone (with the status of a bone growth factor) would be unlikely to possess four potential metal/calcium binding sites in its sequence, if such sites were not involved in the binding of calcium.

Here, we show for the first time ever that calcium does indeed bind quantitatively to β2m, causing conformational changes as a consequence and also bringing about the aggregation and precipitation of β2m into amorphous aggregates that subsequently turn into aggregates with amyloid character (and an appearance of amyloid-like fibrils within a mesh-like aggregate, which bind to amyloid-specific dyes). We also show that this behavior is displayed by the binding of numerous other divalent ions too, including copper. Indeed, we show that the precipitation induced by copper is by far the highest, for all divalent metal ions tested, using comparable metal ion concentrations. However, we also show (and argue) that calcium binding remains the most relevant of all metal binding to β2m because none of the other ions are present in the serum at concentrations comparable to those at which calcium is present.

## Results and Discussion

### Resonance Rayleigh scattering (RRS) indicates that micro-aggregation of β2m occurs at serum-like concentrations of protein and calcium, with reversal seen upon chelation of calcium by EDTA

**Figure 2:**
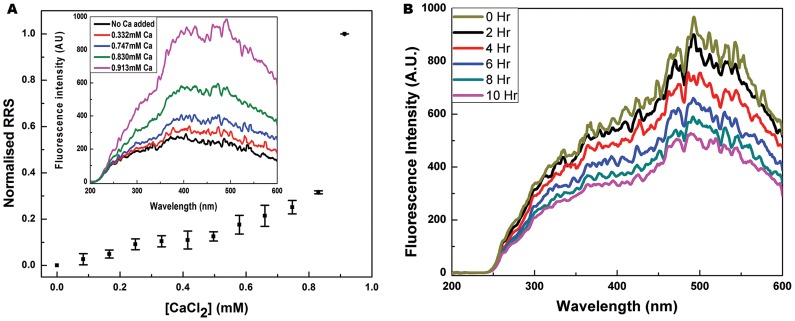
Resonance Rayleigh Scattering data for calcium-induced aggregation of β2m. *Panel A* shows normalised RRS intensities plotted against increasing calcium chloride concentration. The concentration of β2m protein was 0.83 µM, and calcium chloride concentrations were 0.083, 0.166, 0.249, 0.332, 0.415, 0.497, 0.579, 0.66, 0.747, 0.83 and 0.913 mM. The inset shows representative RRS spectral scans at calcium chloride concentrations of 0, 0.332, 0.747, 0.830 and 0.913 mM to illustrate how RRS spectra appear. Peak intensities from such spectra were used for the main plot. *Panel B* shows reduction in RRS signal upon incubation with 10 mM EDTA, at time points of 0, 2, 4, 6, 8, and 10 hours.

A well-accepted method for examining microscopic protein aggregation is to examine whether scattering levels in Rayleigh scattering measurements (see materials and methods) peak in the vicinity of ∼400 nm during collection of RRS spectra, between 200 and 600 nm on a spectrofluorimeter using synchronous scanning of the excitation and emission monochromators, and a Δλ setting of 0 nm [36]. RRS scans essentially plot changes in levels of Rayleigh scattering observed as a function of the wavelength of incident light. In the present instance, we monitored calcium-induced aggregation of β2m at a physiological pH of 7.4, and a physiological temperature of 37°C, using a β2m concentration of ∼0.8 µM which is comparable to that seen in healthy individuals (1–3 µM), and various increasing concentrations of the calcium ion [up to 0.9 mM] well below those observed in the serum of healthy individuals (1–2 mM). Our objective was to examine whether calcium can elicit an increase in the RRS signal in some dose-dependent manner indicative of microscopic aggregation. The inset in Figure 2A clearly shows that RRS spectra obtained at different concentrations of calcium display an increase in Rayleigh scattering in the range of 400 to 500 nm with increasing concentrations of calcium. Representative spectra for four different calcium ion concentrations are shown in the inset, while the main graph in Figure 2A plots changes in the peak RRS signal for different concentrations. The observed increase was non–linear and displayed a clear dose-dependence. Scattering levels increased manifold at ionic concentrations of a few millimolar calcium. This increased scattering is evidence of the presence of micro-aggregated protein and hints at the possibility of β2m aggregation occurring in the serum, since comparable concentrations of β2m were used with lower-than-normal concentration of calcium. While the RRS data is shown for the use of phosphate buffered saline (PBS), entirely similar results were obtained with tris buffered saline (TBS). Therefore, unless otherwise mentioned (where water alone was used, e.g., in isothermal titration calorimetry experiments), for most experiments described below only was used to create physiological conditions. Interestingly, there is also a time-dependent reduction in scattering observed upon addition of EDTA in the resonance Rayleigh scattering data, as shown in Figure 2B.

### Visible turbidity is obtained upon further increase in β2m and calcium ion concentrations

**Figure 3:**
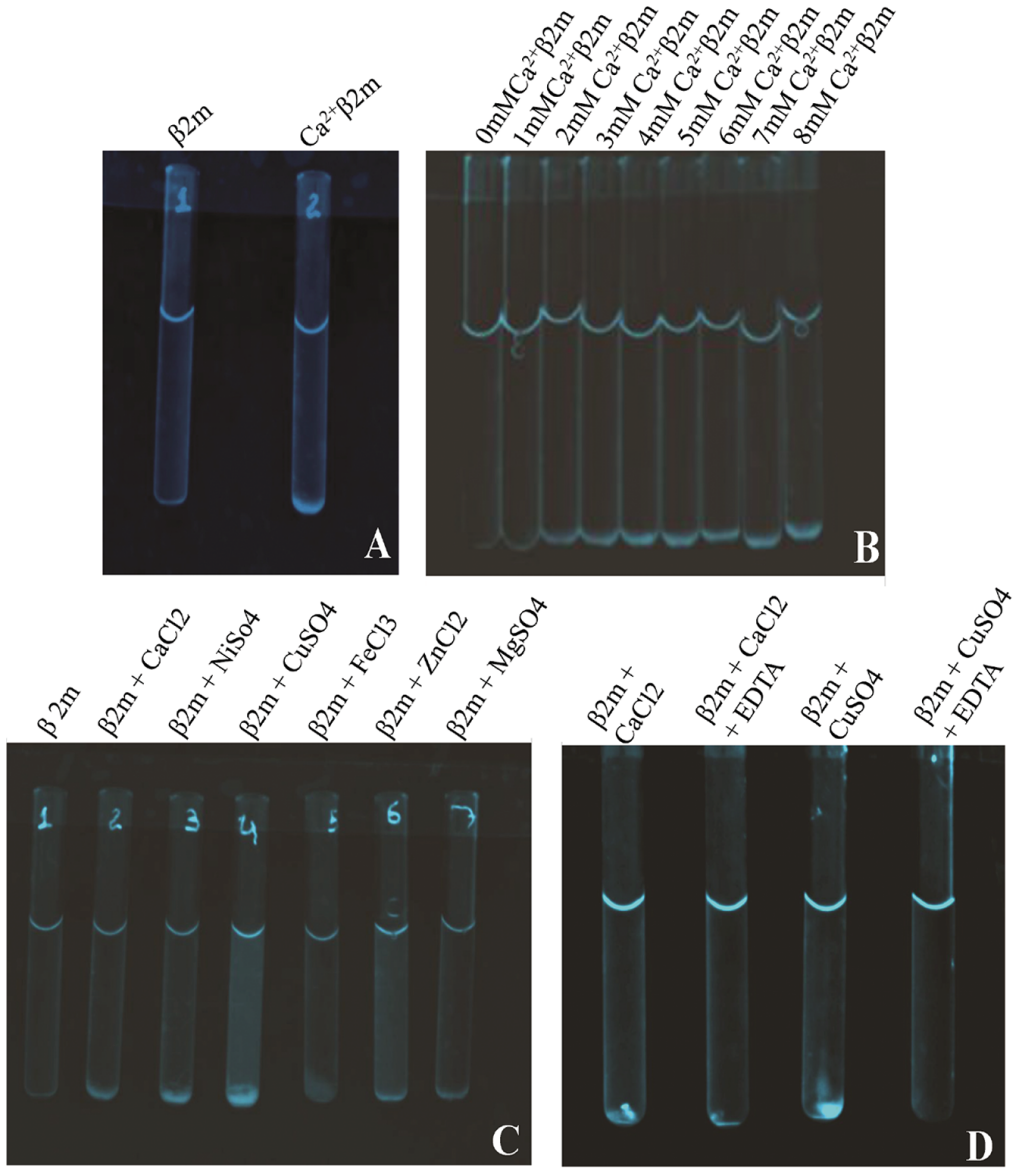
Visual evidence of the role of calcium and other metal ions in causing the aggregation and precipitation of β2m, and the role of EDTA in reversing such aggregation if added immediately after the formation of amorphous aggregates. *Panel A* shows solutions of β2m (control) and calcium-precipitated β2m, respectively, in tubes 1 and 2. For these experiments, a β2m solution (20 µM) was allowed to stand in the tubes for over one hour in the absence (tube 1) and presence (tube 2) of 5 mM calcium chloride, followed by mild centrifugation to sediment the precipitated β2m visible at the bottom of the tube. *Panel B* shows precipitation of β2m in 20 µM solutions by the following different concentrations of calcium chloride: 0, 1, 2, 3, 4, 5, 6, 7 and 8 mM CaCl2, respectively, in tubes numbered 1 through 9, further (visually) establishing the dose-dependence. *Panel C* shows the comparative visible precipitation of β2m in 20 µM solutions by 5 mM concentrations of CaCl_2_, NiSO_4_, CuSO_4_, FeCl_3_, ZnCl_2_, and MgSO_4_, in experiments similar to those shown in previous panels, with the control sample shown in tube 1. *Panel D* shows the formation, deposition and clearance of aggregates of β2m in 20 µM solutions by 5 mM calcium and copper, respectively, in tubes 1 and 3, and the clearance of the same through 24 hours of incubation with EDTA (10 mM), in tubes 2 and 4, respectively.

Figure 3A shows that visible precipitation of β2m is observed within a few tens of minutes of addition of calcium when the concentration of the β2m protein is raised from 4 µM to 20 µM, and that of the calcium ion is raised from below 0.66 mM to 5.0 mM, or above. The identity of the aggregates formed and precipitated was confirmed to be β2m by centrifuging and collecting the aggregated protein and analyzing it on SDS-PAGE by boiling the aggregate with SDS-PAGE loading buffer, to visualize the β2m protein band (data not shown). Figure 3B shows the increase in visible sedimentation of protein obtained as calcium ion concentrations are raised from 0 mM to 8 mM, in 1 mM increments.

### Aggregation and precipitation are also seen with other metal ions

Figure 3C shows evidence of precipitation by a host of different metal ions under entirely similar conditions of buffer pH and concentration and metal ion concentrations (5 mM). It was observed that the greatest amount of precipitation could be obtained with copper. Importantly, the precipitation seen with calcium is comparable to that seen with most other metals. Of course, the important thing is that of all metals for which these experiments are described, only calcium exists in the serum at concentrations (1–2 mM) comparable to those used here (5 mM).

### Visible reversal of turbidity upon addition of EDTA

The visible aggregates that had been formed disappeared within 24 hours of addition of the divalent ion-chelating agent, EDTA, as shown in Figure 3D. This reversal occurred regardless of whether centrifugation had been done (and aggregates re-suspended, prior to calcium addition), or aggregates were still in suspension prior to sedimentation. This observation essentially establishes that the visible aggregation and precipitation of β2m owes to the presence of calcium. It also suggests that there is binding of calcium by β2m, since EDTA could potentially interfere with such binding equilibria by bleeding calcium away and sequestering it. We also examined the relative clearing of aggregates by EDTA for aggregation induced by copper and by calcium, both visually and by RRS measurements.

### Transmission electron micrographs show that aggregates are amorphous when formed

**Figure 4:**
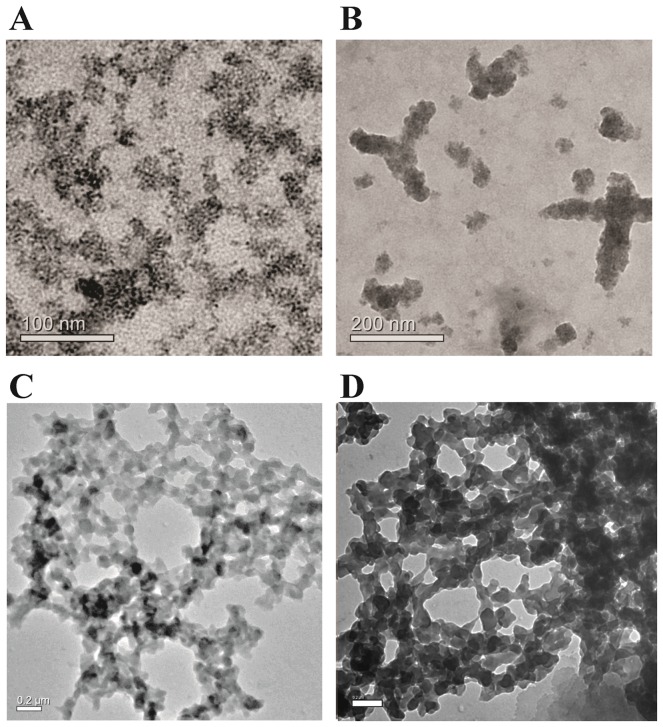
Transmission electron micrographs of amorphous and mesh-like (amyloid-containing?) aggregates of β2m, from experiments such as those described in Figure 3. *Panels A and B*, respectively, show representative views of dispersed amorphous aggregates, and some rare self-organizing aggregates, seen when imaging is done immediately after the aggregation and precipitation of the protein. *Panels C and D*, respectively, show representative views of the networked and branched aggregates that are formed by incubating the amorphous aggregates for three weeks with, or without, periodic shaking. The views in *Panels C and D* cannot be compared with any previously seen amyloid forms, but could represent a pre-fibrillar morphology. The scale bar in *Panel D* which is not very clear is 0.2 µM.

**Figure 5:**
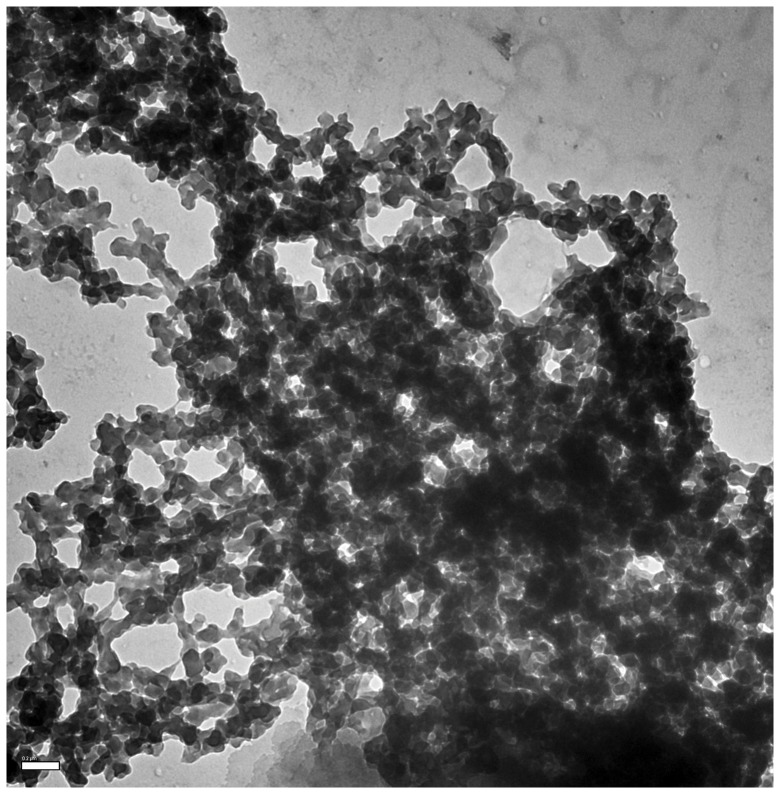
A detailed and representative ‘wide-field’ view covering an area of nearly 3 µm×3 µm is shown for a mesh-like (amyloid-containing?) aggregate of β2m formed without shaking of samples, through three weeks of incubation of amorphous aggregates deposited through calcium-induced precipitation. Within the aggregate, strands (fibrils) varying in diameter from 10 nm to 70–80 nm are seen to network in interconnected fashion.

The morphology of the aggregates was analyzed by transmission electron microscopy (TEM), using standard methods of staining. Figures 4A shows a representative view of the TEM field filled with scattered clusters of aggregates that are clearly amorphous. A few rare representative specimens of somewhat larger (more well delineated) aggregates present in the field are also shown in Figure 4B, hinting at the possibility of some reorganization of the amorphous aggregates into more ordered aggregates. However, there is no evidence of any amyloid-like macrostructure, or the presence of fibrils. *Transmission electron micrographs show amyloid networks after 3*–*4 weeks of incubation.* The amorphous aggregates described above were allowed to remain sedimented in the presence of calcium for 3–4 weeks with, or without, periodic shaking. Two representative micrographs of such incubated aggregates are shown, for aggregates subjected to shaking, in Figures 4C and 4D. One representative micrograph of an aggregate allowed to form without shaking is shown in Figure 5. The morphology of the aggregates appears to have changed considerably after incubation, with a definite ‘branched’ and ‘networked’ pattern of aggregates observed in addition to a somewhat unusual fibrillar character, especially in the parts of the structure that give it a ‘meshwork’ appearance, suggesting that these could be amyloid in nature. The unusual morphology is not of great concern, however, because the morphologies of amyloid aggregates do vary considerably from protein to protein [37] and also for aggregates of the same protein formed under different conditions [6], [38]. In the case of β2m too, as already mentioned, amyloid aggregates have very different morphologies depending on how they were caused to form [6]. Of course, the morphologies of the aggregates reported here are different from the ones formed at acidic pH, or under other conditions, and the modes and mechanisms of formation would also appear to be different. The transformation of the amorphous aggregates into such meshwork-like aggregates with time would be very interesting indeed, if these aggregates were to show any signs of being amyloid-like in nature; this is because this would suggest that amorphous aggregates can act as ‘nurseries’ for the formation of amyloid aggregates. Notably, this is a contention which has previously put forward by other groups, including our own [39], [40]. The hypothesis advanced by Prusiner and colleagues [40] is that either individual molecules, or assemblies of molecules, dissociate from amorphous aggregates and deposit into fibrils or proto-fibrillar structures that are being formed in the vicinity of the amorphous aggregate, through a process of diffusion and readsorption, OR that there is a transformation of chains into proto-fibrillar structures within the amorphous aggregates themselves, and that these then somehow reorganize into progressively more fibrillar morphologies. We have reported [39] that amyloids can form through the assembly of bead-like intermediate structures seen within clumped amorphous aggregates that line-up and transform into amyloid fibers, with some fibers possessing spherical bead-like ends (i.e., displaying evidence of having been generated from bead-like structures). We have also reported the formation of pore-like structures in amorphous aggregates [40] that seem to result from the ‘head’ region of a short amyloid fiber assembling with the ‘tail’ region of the same fiber. Notably, Lindquist and colleagues have also reported that a subpopulation of protofibrils may function as pathogenic amyloid pores [41]. In fact, this group has also suggested in the same paper, and in other publications, that amyloid fibers are a product of the deposition of the real pathogenic (pre-fibrillar) species that are cytotoxic, resulting in a protection of cells from the toxicity of the pre-fibrillar forms. Pre-fibrillar species which do not need to have any clear fibril-like morphology can best be examined through the binding of amyloid-specific dyes. To summarize this section, we wish to emphasize that the unusual meshworked structures observed by us could be pre-fibrillar amyloid forms, with an amyloid-like cross beta sheet structure having already been attained at the level of the reorganization of the polypeptide backbone. The only way of establishing whether this is true would be to examine these aggregates using amyloid-specific dyes like Thioflavin T (ThT).

### Strong Thioflavin T (ThT) fluorescence is seen with aggregates incubated for 3–4 weeks, while none is seen with the amorphous aggregates obtained initially

**Figure 6:**
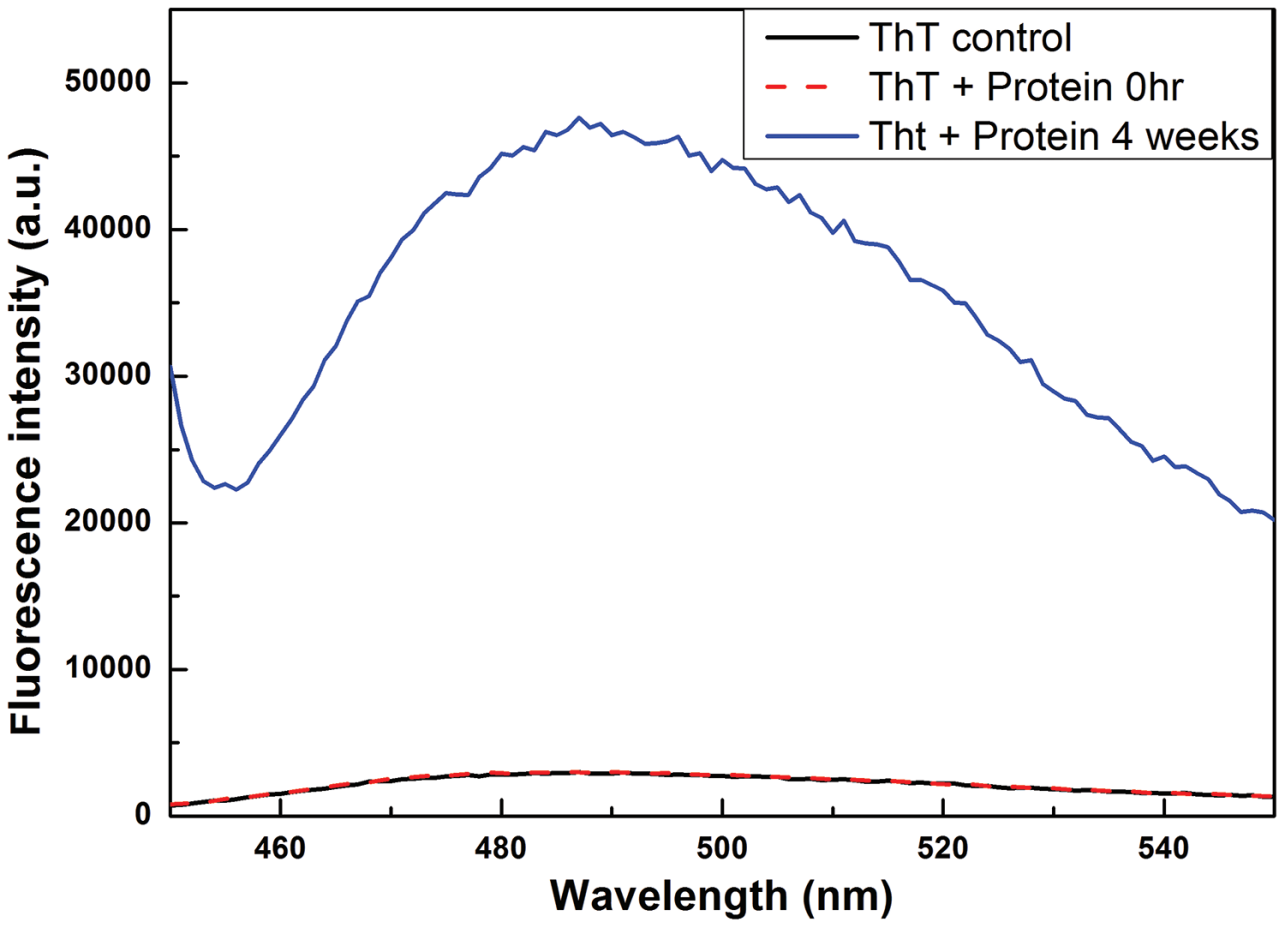
Dye fluorescence spectrum collected with Thioflavin T (ThT) alone (black) and with ThT in the presence of β2m aggregates (red) for aggregates such as those imaged in Figure 5. A profound increase in the emission of ThT is seen to peak at 482 nm (note the break in the y-axis) upon its binding to the cross beta-sheet structure in the aggregates. With amorphous aggregates, no such increase was seen and fluorescence was similar to that seen in control solutions of the dye.

The presence of amyloid-like microstructure in the calcium-induced β2m aggregates incubated for 3–4 weeks (which have undergone transformation from amorphous microaggregates to amyloid-like networks of aggregates) was investigated through examination of the binding of the dye, ThT, to resuspended aggregates placed in the light path of a spectrofluorimetric cuvette, using the characteristic ThT fluorescence seen upon amyloid-binding as a diagnostic criterion. The fluorescence of ThT increases upon amyloid binding. This is a qualitative test, which depends on the quantitation of ThT fluorescence, i.e., the quantum of increase in the intensity of ThT fluorescence depends on the type of amyloid, the relative amounts of the dye and the amyloid, the nature and quality of the resuspension etc. The fluorescence spectra recorded with ThT alone and with ThT added to the calcium-induced β2m aggregates and incubated for 4 weeks, are both shown in Figure 6. There is a clear increase in fluorescence of the dye in the presence of the aggregates, indicating the presence of amyloid-like microstructure. Notably, as Figure 6 shows, no such increase was seen with amorphous aggregates, immediately after their formation and deposition in tubes, whereas ThT fluorescence is seen after the passage of a few weeks.

### Isothermal titration calorimetry (ITC) indicates that β2m binds up to 4 calcium atoms

**Figure 7:**
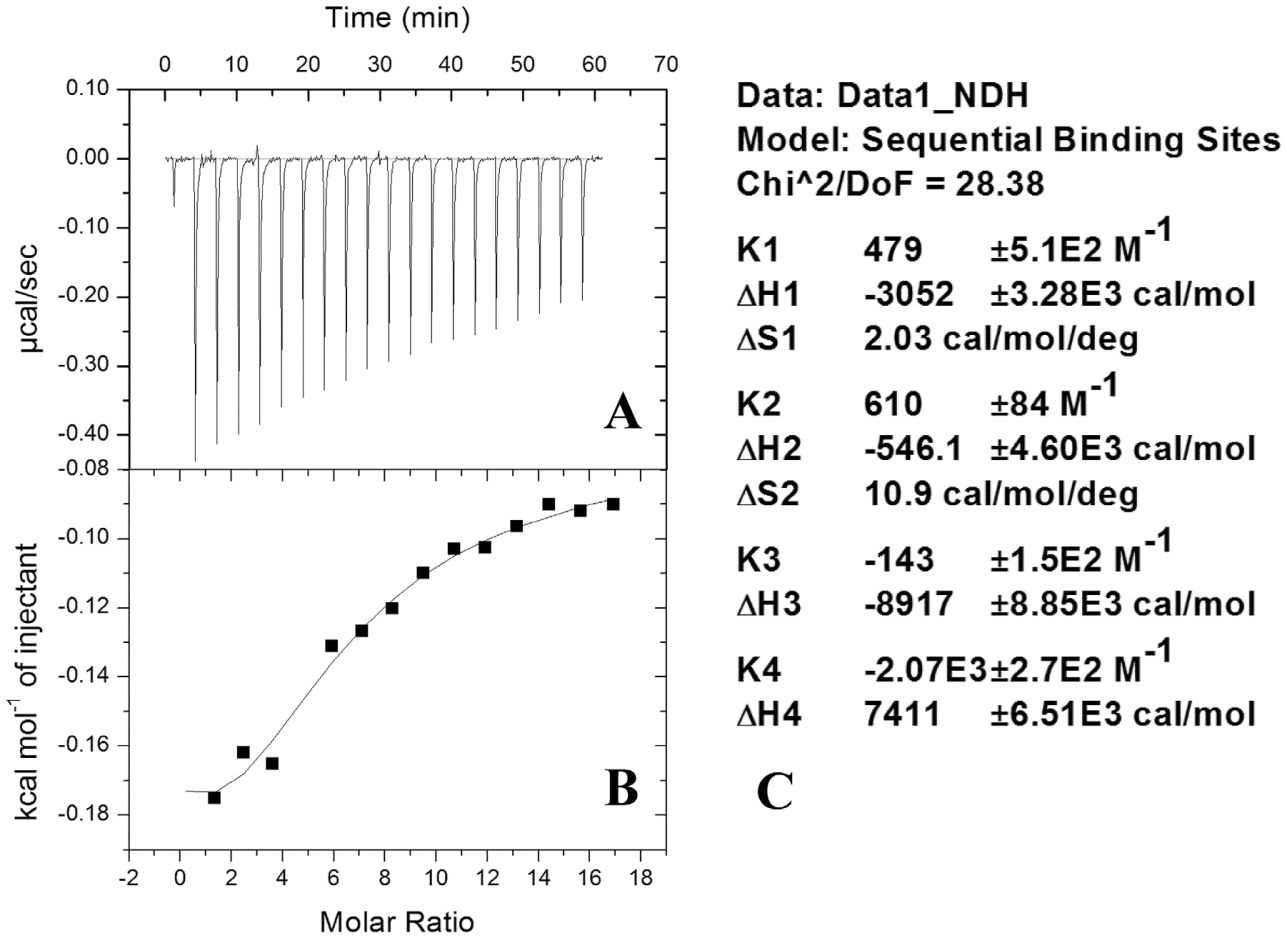
An isothermal titration calorimetry (ITC) thermogram of β2m titrated with calcium chloride is shown in Panel A. The fitting of the data is shown in *Panel B*. The data was best fitted in the sequential-binding model suggesting 4 binding sites. The parameters of the fit are shown in *Panel C*.

The β2m aggregation at higher calcium concentrations can be a consequence of two possibilities. On the one hand, there could be a non-specific ‘bulk’ effect of the presence of calcium in terms of changes in ionic strength which are sensed by the protein, perhaps through some non-specific binding or adsorption of the metal on to the protein's surface. Miranker and colleagues have suggested that there could be such non-specific binding of calcium to the protein [23]. On the other hand, there could be some reasonably specific binding of the calcium to specific sites, or to metal-binding motifs present in β2m, leading to an overall conformational change in the structure and thereby leading to aggregation. By controlling the rate and amount of aggregation, i.e., by using lower protein concentrations, isothermal titration calorimetry could be used to determine whether there is indeed any binding of calcium. An ITC thermogram obtained through titration of calcium chloride (14 mM) into protein (125 µM) is shown in Figure 7A. The fitting of the thermogram is shown in Figure 7B and the parameters obtained for the 4 binding sites along with the assessment of the fitting are shown in Figure 7C. The thermogram suggests specific binding of calcium to β2m. The fitted data suggests sequential binding of the metal ion to the protein at up to 4 sites. Whether these sites are identical to the sites indicated by Vachet and colleagues for copper binding (i.e., the N-terminus and the protein's three histidines in the unfolded state), or whether they are the sites in the sequence that have been pointed out by us (see Figure 1 and the introduction section), of course, remains to be established. Binding constants and other parameters are provided in the box adjacent to the fitted curve. ITC thermograms give a measure of the overall heat change of the system upon binding of two interacting molecules. Here, many changes could be simultaneously taking place, e.g., binding of calcium to metal-binding motifs present on β2m, a resultant conformational change in the structure of the protein, formation of micro-aggregates or oligomers, and finally the aggregation of micro-aggregates into larger amorphous aggregates etc. To investigate this further, we performed FTIR spectroscopy of the protein incubated with calcium as a function of time, since slow sequential binding accompanying the development of turbidity could very well result from an observable time-dependent bulk change in the population's conformation.

### Fourier-transform infra-red spectroscopy reveals marked increase in beta-sheet conformation with calcium binding

**Figure 8:**
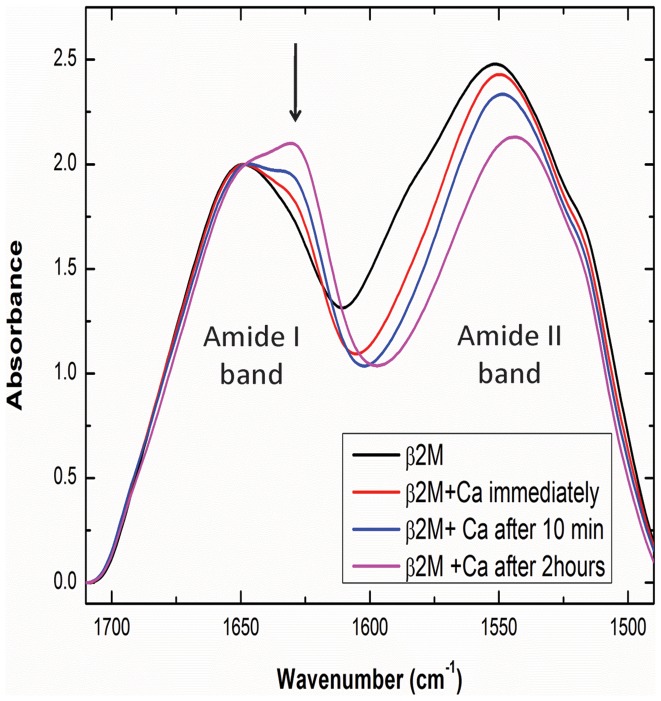
Infra red (HATR-FTIR) spectra of β2m alone (black) and β2m in the presence of calcium, taken immediately after addition of calcium (red), or after the passage of 10 minutes (blue) or 2 hours (pink). Spectra were collected *in situ* on the ATR crystal for samples placed in a sealable (evaporation-proof), temperature-controlled ATR accessory (Bruker Bio-ATR-II) for solutions and any aggregates depositing on the ATR surface during the experiment. In the present instance, the concentrations of β2m and calcium were kept relatively low (see methods) to keep the protein largely in solution in the form of microscopic aggregates and no visible deposition on the ATR surface could be seen. The figure shows the normalized, baseline-corrected amide I and amide II spectral bands of β2m. The amide I band of native β2m (black) shifts very slightly and develops an additional prominence at 1629 cm^−1^ (characteristic of the conversion of some disordered structure into anti parallel beta sheet) in time dependent fashion upon addition of calcium. In the amide II band which is also sensitive to secondary structural changes, the peak shifts gradually from 1550 cm^−1^ to 1540 cm^−1^.

A representative set of Fourier-transform infrared spectra are shown in Figure 8, to establish the effect of calcium binding on the conformation of β2m upon binding of calcium. For this, a solution of the protein was first placed in a conical chamber on the horizontal attenuated total reflectance (HATR) crystal of the FTIR spectrometer, and spectral data was collected for two absorption bands originating in the peptide bond, amide I (1700 to 1600 cm^−1^) and amide II (1600–1500 cm^−1^). Subsequently, calcium was added in a very ‘small volume’ aliquot to the protein, from a highly concentrated stock solution (to prevent any significant dilution effects on the protein, or its FTIR spectrum). Spectral data was collected for the amide I and amide II bands after different time intervals to allow for a ‘phased’ and sequential binding of calcium at different sites in a time-dependent manner. Thus, in Figure 8, spectra were collected immediately before addition of calcium, immediately after addition of calcium, 10 minutes after addition and 2 hours after addition. A single composite band maximum, seen at ∼1660 cm^−1^ in the amide I band envelope of native β2m, transforms into two band maxima. The original envelope with the band maximum at ∼1660 cm^−1^ is constituted of a linear combination of contributions from the beta-sheet signal below 1640 cm^−1^ and the signal from the unstructured component (random coil) which dominates the longer wavenumbers closer to ∼1680 cm^−1^. Against the background of this spectrum, upon addition of calcium, a second band maximum is seen to ‘break through’ the band envelope and become prominent at 1629 cm^−1^, presumably owing to increase in the beta sheet content of β2m at the expense of some unstructured (random coil) regions. The 1629 cm^−1^peak is distinctly visible in the spectrum collected after 2 hours of incubation with calcium. The highlight of this experiment is that the data is collected ‘*in situ*’ on the HATR crystal maintained at a constant temperature, with nothing further being added to the solution, such that the data for solutions incubated for 0, 10, and 120 minutes are all collected on the exact same solution without anything being done to disturb the solution. The data thus owes to both protein in solution, and any settling aggregates coming into contact with the HATR crystal. Satisfyingly, the amide II spectrum, which displays two band maxima at 1550 and 1520 cm^−1^ also shows a shift in the 1550 cm^−1^ peak towards 1540 cm^−1^ upon calcium addition. The amide II band is also sensitive to changes in protein secondary structure, although deconvolution of the band into its component secondary structural contents is not yet technically feasible.

## Conclusions

Whereas it was established nearly nine years ago that the protein, β2m, forms oligomers upon binding to copper, the impression has persisted in the literature - whenever any attention has been paid to this subject at all - that the binding of other divalent ions, such as calcium or zinc, occurs non-specifically. In particular, the binding of calcium to β2m, and the consequences of such binding on the behavior of β2m have not been explored at all beyond the preliminary and cursory examination conducted by Miranker and colleagues [24],[17], which suggested a non-specific binding of calcium, relative to the specific binding obtained with copper.

Here, we have established beyond doubt that calcium causes β2m to undergo aggregation and precipitation, and also that these aggregates are initially amorphous but later turn into amyloid-like forms with ThT-binding characteristics. We have also shown that the aggregates remain microscopic at physiological (serum) concentrations of protein and calcium. However, when concentrations are raised there is a conversion of these microscopic aggregates into very large aggregates that undergo precipitation and deposition. A possibility that arises, therefore, is that the protein exists as microscopic aggregates (large oligomers) under physiological conditions, but that during renal dysfunction (and, in particular, during dialysis) the concentration of these microscopic aggregates rises to untenable levels, leading to the precipitation and deposition of β2m into amorphous aggregates. Perhaps, therefore, it is these aggregates that later turn into amyloids after they have been deposited in bone joints. Perhaps, β2m does not directly deposit in bone joints in amyloid form. Perhaps, therefore, calcium is the culprit that causes physiological deposition of β2m. These are speculations, but worth consideration.

We have speculated that one of the sites of calcium binding could involve a DXEXD calcium binding motif. The motif is conveniently positioned in β2m's sequence and structure at a location that is very close to a loop between two beta strands, making it potentially amenable to conformational alterations of the kind discussed in this paper, which would be necessary for slow and sequential calcium binding at multiple sites. The concept of sequential binding of copper at multiple sites, through conformational changes, has already been broached by Vachet and colleagues [25], [26]. We are postulating different sites for calcium binding owing partly to the observations of Miranker and colleagues that, at low concentrations, copper binding occurs far more spectacularly than calcium binding. We are postulating that calcium-binding could still be specific, and involve a lower affinity as well as a different definition of binding sites, although possibilities concerning some shared sites cannot be altogether ruled out, especially if histidine residues cooperate with the DXD/DXE/EXD motifs for calcium binding. In principle, β2m's DXE or EXD motifs could also potentially bind *in vivo* to sugars, since these exact same motifs are known to be both sugar-binding and metal-binding [31]–[34]. This could explain the correlations between bone metabolism, β2m deposition, and advanced glycation end-products (AGEs) [13]–[15]. Certainly, there are high enough concentrations of sugars in the blood, and in the serum, for β2m to potentially bind to both sugar and metal, utilizing these hitherto unexplored DXD motifs.

We have shown that EDTA reverses the formation of the amorphous aggregates, partially for calcium-binding induced aggregation and almost entirely for copper-binding induced aggregation. Since EDTA therapy is approved by the U.S food and drugs administration for metal-poisoning, it may be reasonable for someone to examine whether such therapy given immediately before and after dialysis can prevent, or reduce, the DRA. Certainly, given the fact that EDTA is approved for therapy, a regimen of therapy involving lower concentrations that those recommended for metal-poisoning cases could be worked out.

An interesting possibility arising from this data concerns the possible degradation of the β2m aggregates even before their degradation in the kidney. Some years ago, Sharma *et al* showed very elegantly, and with great rigor, that aggregates formed by a variety of proteins at near-neutral pH bind to metal ions and utilize serine residues on their surfaces to carry out metal-catalyzed proteolysis that results in the ‘self’ or auto-degradation of these proteins in solution [42]. Of course, in the present instance, it is not as if calcium binds to the β2m aggregate after its formation. Rather, metal (calcium) binding is responsible for the formation of the aggregate. Still, it is conceivable that as β2m microaggregates circulate in the blood under normal conditions, some degradation into peptides and amino acids takes place. Of course, more work will be required to examine whether this can indeed occur, both *in vitro* and *in vivo*.

A remarkable further aspect of this study relates to the correlations that can be drawn between calcium and β2m, not so much in relation to disease and protein precipitation under the extremely unusual conditions brought about through dialysis, but rather in terms of the role of such binding under normal healthy conditions in a human being. One would presume that if calcium binding to β2m is not non-specific (i.e., if it is specific), nature would have designed β2m to be capable of binding to metals for some purpose. It is true that the discovery of copper binding to β2m could originally have given rise to this question, many years ago. Still, since copper concentrations are never high enough *in vivo*, the question of a natural purpose did not somehow arise at that time. With the publication of this work, of course, the question of ‘purpose’ does arise, given calcium's concentrations, and roles, in the human body and in metabolism. We have shown that metal-binding causes aggregation and precipitation of β2m, and also shown that calcium binding and β2m precipitation occur at physiological concentrations of protein and metal. So, it is time to ask what the purpose of this binding could be.

One purpose could be that calcium-binding by β2m is used by calviariae to deplete osteoclasts and osteoblasts, allowing β2m to function as a mitogen and growth factor. In other words, the importance of β2m to bone growth and metabolism could potentially be mediated not through the binding of β2m to some protein receptor on cells (which would be unrelated to calcium), but directly through the sequestration of calcium.

Another purpose could involve the auto-degradation of ‘shed’ β2m molecules forming micro-aggregates in the serum, outside the kidney, as already elucidated above.

A third hitherto unsuspected purpose could be to stabilize β2m binding to the HLA chain in MHC complexes. This aspect has never been explored, but it is certainly worth exploring, for the following reasons. The reported negative correlation between serum calcium levels and serum β2m levels, alluded to in the introduction section of this paper, could play out in two different ways, as far as ‘cause-effect’ relationships are concerned. On the one hand, more serum β2m could result in less serum calcium on account of shed β2m acting as a sink for calcium in the serum (i.e., in addition to the ‘calcium sink’ roles played by other proteins such as albumin). On the other hand, if binding of calcium by β2m (even at one site, e.g., the DXEXD site) is required for β2m to remain bound stably to the HLA chain in MHC-I complexes, it is conceivable that a lowering of serum calcium concentrations (for some unconnected reason) could result in a deficiency of calcium availability for β2m binding, resulting in greater shedding of β2m.

## Materials and Methods

### β2m expression

A clone of human β2m with a C-terminal 6xHis tag, sub-cloned in the pET 23A vector (between a 5′ -NdeI site and a 3′ -XhoI site) was overexpressed in, and purified from, *E.coli* cells of the BL21 Star (DE3) pLysS strain. Transformed cells were grown overnight at 37 °C with 100 µg/ml ampicillin and 35 µg/ml chloramphenicol. An overnight culture was sub-cultured into 500 ml of LB broth in a 1.0-liter flask containing the same antibiotics and cells were grown at 37 °C in a rotary shaker until the culture reached an OD_600_ of 0.6. Protein expression was then obtained through induction by 1 mM IPTG, with induced cultures being grown overnight. Cells were pelleted through centrifugation at 5000 rpm for 10 minutes and treated as given below.

### β2m purification under denaturing conditions

Pelleted cells containing overexpressed β2m protein were re-suspended in 100 mM NaH2PO4, 10 mM Tris-Cl, 8 M urea, pH8 (50 µl per ml of culture), and sonicated to effect cell lysis. The supernatant was separated from cell debris through centrifugation at 16,000 rpm for 20 minutes at 4 °C. Purification was achieved by loading the supernatant onto a Ni-NTA affinity column (1 ml resin, Qiagen) pre-equilibrated with the sonication buffer. Non-specifically bound proteins were removed by washing with 40 ml of wash buffer (100 mM NaH2PO4, 10 mM Tris-Cl, 8 M urea, pH 6.5). The bound 6xHis tagged protein was eluted using standard elution buffer (100 mM NaH2PO4, 10 mM Tris-Cl, 8 M urea, pH 4.5).

### β2m refolding and reconstitution

The eluted β2m was reconstituted by extensive dialysis against deionized water to remove urea, followed immediately by dialysis against either Tris-buffered saline (TBS), or phosphate-buffered saline (PBS) of progressively decreasing pH values of 8, 7.8, 7.6 and 7.4, to obtain protein in physiological buffers of pH 7.4. It may be noted that this series of dialysis steps is critical. If eluted protein is directly dialyzed against TBS or PBS without first being dialyzed against water to remove urea, there is extensive protein precipitation observed; however, no precipitation whatsoever is observed when dialysis is initially carried out against deionized water (with a pH of ∼6.0) and followed by progressively dialysis against TBS or PBS, initially using mildly alkaline pH before gradually reducing the pH to 7.4. The TBS used had the following composition and characteristics: 25 mM Tris, 150 mM NaCl, 2 mM KCl, pH 7.4. Similarly, the PBS used had the following composition and characteristics: 137 mM NaCl, 2.7 mM KCl, 10 mM Na_2_HPO_4_, 1.8 mM KH_2_PO_4_. It may be noted that for isothermal titration calorimetry (ITC) experiments, TBS or PBS were not used; instead, eluted β2m was extensively dialyzed against deionized water with several changes of deionized water, and this protein was used. TBS or PBS buffers were not used for ITC experiments because of problems with the heat of dilution observed in mixing buffered solutions of protein with buffered solutions containing metal ions. The dialyzed protein was concentrated using Amicon centrifugal concentrators with 3000 Dalton cutoffs.

### Experiments with calcium addition

#### Resonance Rayleigh Scattering (RRS)

A Cary Eclipse spectrofluorimeter (Varian) was used to measure RRS spectra and intensities using a cuvette with a path length of 1 cm. The RRS spectrum was collected by synchronously scanning excitation and emission monochromators between 200 to 600 nm, using a wavelength difference (Δλ) of 0 nm, and monochromator bandpass values of 5 nm each. The concentration of β2m protein used was 0.83 µM and the following concentrations of calcium chloride were used to monitor the RRS signal: 0.083, 0.166, 0.249, 0.332, 0.415, 0.497, 0.579, 0.66, 0.747, 0.83 and 0.913 mM respectively. In separate experiments examining the effect of EDTA addition on the RRS signal of pre-formed aggregates, we added 10 mM EDTA and monitored the drop in RRS signal for 10 hours (with an RRS spectrum being collected after every 30 minutes of incubation). While plotting this data, we reduced the data density and plotted only RRS spectra collected every 2 hours.

#### Horizontal Attenuated Total Reflectance (HATR) Fourier Transform infrared (FT-IR) spectroscopy

FTIR spectra of β2m (20 µM) were measured in TBS, using a Tensor 27 spectrometer equipped with the sealable, temperature-controlled Bio-ATR-II protein sample accessory and CONFOCHECK software. For this, a 25 µl volume of β2m solution was placed in a conical chamber associated with the HATR crystal, and the control spectrum for the protein was collected. Following this calcium chroride was added (final concentration 125 mM) and spectra were collected either immediately, or after the passage of 10 minutes and 2 hours, respectively, to monitor spectral changes indicative of structural changes, if any, in the protein. Any microscopic aggregates would have settled onto the crystal's surface and contributed to the spectrum during the experiment. In any HATR crystal, the absorption signal only owes to the layers of molecules immediately proximal to the crystal's surface (and within the distance accessed by the evanescent wave associated with the FTIR beam undergoing total internal reflection in the crystal). Thus, spectra collected at later time points could owe to a combination of molecules in solution and any depositing aggregates, although no visible deposition was seen, owing to the low calcium concentration used.

#### Isothermal Titration Calorimetry (ITC)

ITC experiments were done using an ITC 200 instrument (GE-Microcal). Purified β2m was extensively dialyzed against MilliQ deionized water with ten changes. The dialyzed protein was filtered through a 0.22 µm filter. Calcium chloride was also dissolved in the same miliQ water used in final change during dialysis, to reduce effects due to heat of dilution due to ITC. Calcium chloride (14 mM) solution was injected into the sample cell (different injection volumes as mentioned below) containing 200 µl of β2m solution (125 µM). The titration was done at 25 °C with an initial 0.4 µl injection followed by 19 injections of 2 µl each, with 180-second intervals between each injection. The data were plotted as a function of molar ratio and binding isotherms were fitted using Origin 7.0 software provided with the instrument.

#### Transmission Electron Microscopy (TEM)

20 µM β2m in TBS containing 4 mM CaCl_2_ in TBS was incubated to create precipitates. The precipitates were spread out on grids, and negatively stained with phosphotungstic acid (PTA) and imaged using standard protocols on a JEOL JEM-2100 microscope.

#### Thioflavin T fluorescence

Fluorescence spectra of thioflavinT (ThT) dye controls, amorphous protein aggregate controls, as well as dye bound to amyloid-like protein aggregates, were collected on a Horiba Fluoromax-4 spectrofluorimeter, with the excitation monochromator set at 440 nm and emission collected between 450 and 550 nm, using with bandpasses of 2.5 nm, and 5 nm, respectively. Samples were prepared in deionized water. The protein sample containing 20 µM β2m, 4 mM CaCl_2_ and 12.5 µM ThT was incubated for three weeks at 37 °C with shaking.

### Visual examination of precipitation

#### Precipitation with other metals

The ability of metal ions to precipitate β2m was assessed visually by monitoring the amount of precipitate obtained, at various intervals of time. Tubes containing precipitates were photographed. Stock solution of CaCl_2_, NiSO_4_, CuSO_4_, ZnCl_2_, FeCl_3_, MgCl_2_, were prepared in Tris buffered saline (pH7.4) and filtered through 0.2 µm filter and added to final concentration of 5 mM, in 20 µM β2m presen in Tris buffered saline (pH7.4).

#### Effects of using different concentrations of calcium

Various concentrations of calcium (0.5 to 5.0 mM) were incubated with 20 µM solutions of β2m protein, in different tubes. Photographs were taken of the extent of precipitation.

#### Disassembly of aggregates with EDTA

Tubes containing precipitated protein (containing 5 mM calcium chloride) were incubated with 5 mM or 10 mM EDTA for 24 hours to monitor dissolution. Photographs were collected with appropriate controls.
